# Retrospective comparative study of rigid and flexible ureteroscopy for treatment of proximal ureteral stones

**DOI:** 10.1590/S1677-5538.IBJU.2015.0644

**Published:** 2016

**Authors:** Ehab Mohamad Galal, Ahmad Zaki Anwar, Tarek Khalaf Fath El-Bab, Amr Mohamad Abdelhamid

**Affiliations:** 1 Department of Urology, Minia University, Minia, Egypt

**Keywords:** Calculi, Ureteroscopy, Ureter, Lithotripsy

## Abstract

**Background:**

We analyzed the outcome and complications of rigid (R-URS) and flexible (F-URS) ureteroscopic lithotripsy for treatment of proximal ureteric stone (PUS).

**Subjects and methods:**

Retrospective data of 135 patients (93 males and 42 females) submitted to R-URS and F-URS for treatment of PUS in the period between July 2013 and January 2015 were investigated. (R-URS, group 1) was performed in 72 patients while 63 patients underwent (F-URS, group 2).We compared the 2 groups for success, stone characteristics, operative time, intraoperative and postoperative complications.

**Results:**

The overall stone free rate (SFRs) was 49/72 (68%) in group 1 and 57/63 (91%) patients in group 2, (P=0.005). The operative time was shorter in group 1 in comparison to group 2 with statistically significant difference (P=0.005). There was not any statistically significant difference between 2 groups in complication rate (P=0.2).

**Conclusıon:**

Both R-URS and F-URS could be a feasible option for treatment of PUS. R-URS is less successful for treatment of PUS and should be used cautiously and with availability of F-URS.

## INTRODUCTION

Based on the findings of European Association of Urology (EAU) Nephrolithiasis Guidelines, shock wave lithotripsy (SWL) and ureteroscopy (URS; retrograde or antegrade) remain the primary treatment modalities for the management of symptomatic proximal ureteral stone (PUS) with comparable stone-free rates (SFRs) (1, 2). Certainly, SWL does offer potential advantages over ureteroscopy in the treatment of PUS <10mm; this is likely due to shorter convalescence and the lack of requirement for anesthesia during the procedure (3). The advances in flexible ureteroscopy and intracorporeal lithotripsy especially holmium laser have revolutionized the treatment of PUS with higher success and lower complication rates (4). All treatment modalities were investigated for SFRs and complications after management of PUS, however to the best of our knowledge, there is only one published article comparing rigid URS (R-URS) and flexible URS (F-URS) for the treatment of PUS (5). We analyzed the outcome and complications of rigid (R-URS) and flexible (F-URS) ureteroscopic lithotripsy for treatment of proximal ureteric stone (PUS).

## MATERIALS AND METHODS

The records of 135 patients (93 males and 42 females) submitted to R-URS and F-URS for treatment of PUS in the period between July 2013 and January 2015 were investigated retrospectively. R-URS (group 1) was performed in 72 patients while 63 patients underwent F-URS (group 2). PUS was defined as the stone located between the superior margin of the sacroiliac joint and the ureteropelvic junction. Eligible patients were ≥20 years of age, who were operated for solitary PUS using URS and had postoperative data regarding SFRs. The exclusion criteria included patients with multiple stones, previous ureteral or renal surgery on the same side and any associated ureteral pathology. All patients were subjected to preoperative evaluation including detailed surgical history, basic laboratory and radiologic investigations including renal function tests, urine analysis and urine culture, plain X-ray of the kidneys, ureter and bladder (KUB) in addition to another radiographic study from among the following: ultrasound, excretory intravenous urogram (IVU), non-contrast computerized tomography (CT). Ureteroscopy was indicated or preferred by our patients due to failed SWL, obesity, and patient preference. Follow-up of the patients was done for a minimum of 3 months. A KUB film was obtained in the immediate postoperative period and urinary ultrasound or CT 3 months later. Perioperative complications were recorded according to the Clavien-Dindo classification system (6).

### Procedures

URS was done under spinal or general anesthesia; patients were given intravenous 200mg ciprofloxacin one hour before induction. Patients were positioned in the dorsal or low lithotomy position, and preliminary cystoscopy was done using 22 or 17Fr cystoscope then a 0.038 inch floppy-tipped guidewire was passed through the selected ureteric orifice, advanced under direct vision, and monitored fluoroscopically. The ureteroscope advanced in the ureter alongside the guidewire. In all cases, we used a 6.5/8.5F (R-URS) (Richard Wolf, Knittlingen, Germany). Once the stone had been visualized, disintegration was done using 20W holmium: YAG laser (Lumenis, Santa Clara, CA, USA). A 200-μm laser fiber with an energy output of 0.8–1.5 joule at 8–12 hertz was used. Residual fragments >2mm were extracted using 1.6F zero-tipped nitinol stone basket (Cook Medical, Bloomington, IN, USA). At the end of the procedure, we evaluated carefully the ureteral lumen for any complications and then 4.8F 26cm double j stent (DJS) was inserted based on surgeon's decision and removed under local anesthesia. When the stone migrated to the kidney, the procedure was completed using F-URS or DJS inserted for SWL according to stone size and exact site inside the collecting system while rigid ureteroscopy was accepted as unsuccessful. These patients were not included in group 2. In case of inability to advance the ureteroscope even after trial of ureteral dilation, a DJS was inserted into the ureter and second look was done at least after 2 weeks, and the procedure was also accepted as unsuccessful.

F-URS was done using 7.5F flexible ureterorenoscope (Karl Storz, Tuttlingen, Germany). After passing a 0.038 inch floppy-tipped guidewire through the cystoscope under fluoroscopy, a Flexi-Tip Dual Lumen Ureteral Access Catheter (Cook Medical, Bloomington, IN, USA) was used for insertion of a second 0.038 inch guidewire. A ureteral access sheath (9/11F or 12/14F) was introduced over the guidewire. We inserted F-URS into the ureter and disintegration was done using 200-micron holmium laser fiber at an energy level of 0.6–0.8J and at a rate of 10–25Hz. Residual fragments >2mm were extracted using 1.6F zero-tipped nitinol stone basket and a 4.8F 26cm DJS was left in place after careful evaluation of the ureteral lumen for any complications. Unsuccessful procedure was considered when we could not pass the access sheath even with trial of ureteral dilation.

### Statistical analysis

Results are presented as mean±standard deviation (SD). Statistical analysis was performed using the Statistical Package for Social Sciences version 20.0 software (SPSS Inc., Chicago, IL). Student's t-test and chi-square test were used for statistical analysis. A value of P <0.05 was considered as statistically significant.

## RESULTS

On retrospective analysis of study data, there were 56 (77.8%) males, 16 (22.2%) females in group 1, and 37 (58.7%) males, 26 (41.3%) females in group 2 (p=0.02). The mean patient age was (38.6±11.2) in group 1 and (39.4±11.8) in group 2 with no statistical significance (P=0.7). Stone demographics (side, size and opacity) were not statistically significant between the two groups (P=0.4). The mean operative time was calculated from the time of cystoscope to the end of stent placement. It was shorter in group 1 in comparison to group 2 with statistically significant difference (P=0.005) (Table-1). The mean hospital stay (P=0.8) and use of DJS (P=0.143) were not statistically significant between the two groups.

**Table 1:** Demographic and clinical characteristics of study groups.

Variable		Group 1(R-URS)	Group 2(F-URS)	p value
		N=72	N=63	
Gender (M/F)**		56/16	37/26	0.2
Mean patient age (year)*		38.6±11.2	39.4±11.8	0.7
Mean ureteral stone diameter (mm)*		13.5±3.5	12.9±2.8	0.3
**Laterality** **				
	Right	39 (54.2%)	30 (47.6%)	0.4
	Left	33 (45.8%)	33 (52.4%)	
**Stone Opacity** **				
	Radioopaque	41 (56.9%)	31 (49.2%)	0.4
	Radiolucent	31 (43.1%)	32 (50.8%)	
Mean operative time (min)*		40.9±16.4	48.4±13.8	0.005

* Student t-test (P <0.05)

** Chi-square test (P <0.05)

Procedures were successfully completed in 49 (68%) patients in group 1 and 57 (91%) patients in group 2. This value was statistically significant (P=0.005).

Failure in group 1(n=23) was due to inability to advance the ureteroscope to the level of stone due to stone impaction with associated ureteral edema (n=7), stone migration (n=9) 3 of them were completed using F-URS while DJ inserted in other 6 patients for further SWL. In 2 patients, the procedure was aborted due to mild ureteral perforation and DJ stent was inserted. Five patients were considered unsuccessful in early follow-up due to large residual stone fragments that needed secondary procedure.

In patients of group 2 (n=6) with unsuccessful access to the stone, we could not pass the access sheath due to narrow ureteral lumen even after trial of dilation. In all cases 4.8/26 DJ stent was left for 15 days.

There were no major intraoperative complications (grade 4 or 5) according to Clavien-Dindo classification system (6) in either group. Ureteral perforation (grade 3B) was observed in 2 patients in group 1 and managed as mentioned. The early postoperative complications were compared and there was not any statistically significant difference between two groups (P=0.2). Hematuria was observed in 15 (21%) patients in group 1 and 11 (17%) patients in group 2 while, renal colic and fever occurred in 4 (5.5%) and 3(4.7%) patients in group 1 and 2 respectively with only one patient in group 1 had documented urinary tract infection.

Of the 49 patients in group 1 cleared of stones during follow-up, 39 (79.5%) patients were improved with evidence of radiological patency and reduction or disappearance of perioperative dilatation in comparison to 54 (94%) patients in group 2 and this value was statistically significant (P=0.006) (Table-2).

**Table 2:** Success rate, operative and postoperative data of study groups.

Variable	Group 1(R-URS)	Group 2 (F-URS)	p value
	N=72	N=63	
Success rate**	49 (68%)	57 (91%)	0.02*
Intra-operative complications**	25%	9.5%	0.3
Postoperative complications**	19 (26.38%)	14 (22.2%)	0.2
Follow-up success**	79.5%	94%	0.006
Hospital stays (hours)*	18.3±8.3	18.6±7.8	0.8

* Student *t*-test (P <0.05)

** Chi-square test (P <0.05)

## DISCUSSION

Although SWL is the standard treatment for solitary PUS, still the urologists need to discuss with the patients other treatment options. SWL has the advantages of less invasiveness with stone free rates that range from 80% to 88% (7). However, increasing body mass index, contraindications in pregnancy and patients with bleeding disorders are limitations for SWL (8). Most of our patients preferred URS as the therapeutic modality may be due to stone free status with lower retreatment rate. The development of small-caliber, semirigid ureteroscopes and intracorporeal lithotripsy have revolutionized the treatment of PUS. In a study done by Atis and colleagues, successful outcome with R-URS was achieved in 25 out of 47 patients with renal pelvic stones using holmium:YAG laser (9). Some studies demonstrated wide range of success of (R-URS) (10, 11). Tunc et al. (12) reported their experience in the management of ureteral stones with 60% success rate in the upper ureter for a mean stone size of 12.87mm. In our study, (R-URS) SFRs, mean stone size, and mean operative time were 68%, 13.5±3.5 and 40.9±16.4 respectively, which was comparable to the literature. In a recent study from Korea, success rate was decreased with stone size ≥10mm (13). Other studies showed higher success rate. Sofer et al. (14) reported 97% success rate for PUS, with mean stone size 11.3mm. This may be attributed to the experience of this group in treating proximal ureteral stones and technical armamentarium of the clinics. In our series, the failure rate with R-URS was 32% which was much higher than others, due to increased number of stone migration (n=9). In our opinion, if we had stone-cone® or N-Trap® basket in the clinics of our hospital, the success rate of R-URS group would be higher. The SFRs for the treatment of the patients having PUS was significantly increased by using the F-URS. Furthermore, the use of an access sheath increases the ease of passing the ureteroscope, minimizing intrarenal pressures, facilitates clear vision for the surgeon and allowing removal of large stone fragments (15). With URS lithotripsy using holmium: YAG laser, the SFRs of upper ureteral stones are around 87%-97% (16, 17). In our study we reported similar results as F-URS was performed in 63 patients with SFRs achieved in 57 (91%). The overall success rate of F-URS group was statistically higher than R-URS group and in our opinion, except in 6 patients with narrow ureteral lumen, these were because we did not face with any difficulties in application of the access sheaths over guide wires, so that obviate the factors interfering to reach the stone like tortuous ureter and edema at the site of the stone.

In our study, intraoperative complications occurred in group 1 were statistically higher than group 2, (25% versus 9.5% respectively) (P=0.3). Most of complications were related to failure of access in 7 and 6 patients in group 1 and 2 respectively. Stone migration occurred only in group 1 (12.5%) and this may be explained by increased irrigation to improve visibility during fragmentation using R-URS which usually is not needed in group 2 due to use of access sheath. Manohar et al. (18) reported 24% stone migration, while El Ganainy et al. (19) reported 9%. In two (2.7%) patients of group 1, we had mild ureteral perforation and DJ stent was inserted for 6 weeks. They underwent intravenous pyelography two weeks after removal of ureteral stent with no evidence of ureteral stricture or extravasation. In one series, ureteral perforation was reported in 11 (3.2%) cases (13). While, in another study of Karadag et al. (5) a 2cm ureteral perforation occured with F-URS in one (1.6%) patient and managed with DJ stent for 6 weeks. No statistically significant difference was found between the groups regarding postoperative complications. Hematuria was observed in 15 (21%) patients in group 1 and 11 (17.4%) patients in group 2 while renal colic and fever occurred in 4 (5.5%) and 3 (4.7%) patients in group 1 and 2 respectively. Third month radiologic investigations revealed 39 (79.5%) patients were improved with evidence of radiological patency and reduction or disappearance of perioperative dilatation in comparison to 54 (94%) patients in group 2 and this value was statistically significant (P=0.006) and this showed us the superiority of F-URS in terms of success.

There are some limitations of our series. It was retrospective conducted at a single center, presence of more than one urologist with different experience and surgical skills. Randomized prospective larger series with long period follow-up are necessary to confirm the effectiveness of rigid versus flexible ureteroscopic lithotripsy for PUS.

## Conclusions

Both R-URS and F-URS could be a feasible option for treatment of PUS. R-URS is less successful for treatment of PUS and should be used cautiously and with availability of F-URS. The higher success and lower complications rate of F-URS make it the first line favorable option for treatment of PUS.

## Abbreviations

PUS: = Proximal ureteral stone
MET: = Medical expulsive therapy
ESWL: = Extracorporeal shock wave lithotripsy
URS: = Ureteroscopy
PCNL: = Percutaneous nephrolithotomy
LAP: = Laparoscopy
AUA: = American Urological Association
EUA: = European Association of Urology
R-URS: = Rigid URS
F-URS: = Flexible URS
SFRs: = Stone free rates
BMI: = Body mass index
DJS: = Double J stent
CT: = Computed tomography
US: = Ultrasonography
